# Factors Influencing Cortisol Concentrations in Breastmilk and Its Associations with Breastmilk Composition and Infant Development in the First Six Months of Lactation

**DOI:** 10.3390/ijerph192214809

**Published:** 2022-11-10

**Authors:** Monika A. Zielinska-Pukos, Joanna Bryś, Natalia Kucharz, Agnieszka Chrobak, Aleksandra Wesolowska, Iwona Grabowicz-Chądrzyńska, Jadwiga Hamulka

**Affiliations:** 1 Department of Human Nutrition, Institute of Human Nutrition Sciences, Warsaw University of Life Sciences—SGGW, Nowoursynowska St. 159, 02-776 Warsaw, Poland; 2 Department of Chemistry, Institute of Food Sciences, Warsaw University of Life Sciences—SGGW, Nowoursynowska St. 159, 02-776 Warsaw, Poland; 3 Hirszfeld Institute of Immunology and Experimental Therapy, Polish Academy of Sciences, 53-114 Wrocław, Poland; 4 Laboratory of Human Milk and Lactation Research at Regional Human Milk Bank in Holy Family Hospital, Department of Neonatology, Faculty of Life Sciences, Medical University of Warsaw, Litewska 14/16 Str., 00-575 Warsaw, Poland; 5 Psychological-Pedagogical Counselling Centre No 12, Dzielna St. 1a, 00-162 Warsaw, Poland

**Keywords:** human milk, glucocorticoids, anthropometric development, temperament, psychomotor development, macronutrients, fatty acid profile, body mass index

## Abstract

Previous studies provided contradictory results regarding the influence of maternal, seasonal, and infant factors on breastmilk cortisol, and its associations with breastmilk composition and infant development. This study aimed to assess breastmilk cortisol levels at the first, third, and sixth months of lactation and evaluate the associations with maternal psychosocial, seasonal, and infant factors, breastmilk composition, and infant anthropometric and psychomotor development and temperament. Cortisol concentrations were assessed by ELISA in 24 h breastmilk samples obtained from 38 healthy mothers. Maternal psychological status was assessed by EPDS and PSS-10 and infant psychomotor development was assessed using the Children’s Development Scale (DSR). Breastmilk cortisol was 11.2 ± 6.2, 11.2 ± 4.3, and 12.7 ± 6.2 ng/mL at the first, third, and sixth months of lactation (*p* > 0.05), respectively. In the spring-summer season, we observed lower and higher levels of cortisol in the first and sixth months of lactation (*p* ≤ 0.05), respectively, but no other associations were detected regarding maternal or infant characteristics. In the third month of lactation, cortisol was related to breastmilk crude protein (β = 0.318, 0.007–0.630) and infant BMI z-score before adjustment for infant birthweight and sex (Model 2: β = 0.359, 0.021–0.697), but no other associations with breastmilk composition, infant development, or temperament were confirmed. Our results indicated that breastmilk cortisol is unrelated to maternal and infant factors and has limited influence on breastmilk crude protein, but not on infant anthropometric and psychomotor development.

## 1. Introduction

Breastmilk is the gold standard in infant nutrition and provides not only all necessary nutrients, but also a variety of bioactive compounds that support healthy infant growth and development [1,2,3]. Among many of the bioactive factors, breastmilk contains glucocorticoids (GCs): cortisol, cortisone (an inactive cortisol metabolite), and corticosterone [4,5,6,7,8,9,10,11]. The predominant breastmilk glucocorticoid is cortisone [6,7], followed by cortisol (highly correlated with cortisone levels) [4,6,7,10], and corticosterone, which occurs in the lowest concentrations [11]. Interestingly, cortisol is the predominant GAs in serum, but it is converted to cortisone before being transferred to the saliva and breastmilk [4,12]. Thanks to lipophilic properties, GCs can cross the mammary gland epithelia through simple diffusion [8], as systemic circulation is probably their main source, as breastmilk cortisol reflects diurnal variations related to maternal HPA axis activity [6,7,8] and are highly correlated with serum [5,13] and saliva [6,13] GCs. Despite those lipophilic properties, milk GCs are mainly associated with the skim fraction, probably due to binding to proteins, e.g., corticosteroid-binding proteins, globulin, and albumin [8,9].

As breastmilk GCs reflect their serum levels, it was hypothesized that breastmilk GCs are an important biochemical signal of environmental conditions from mother to infant. According to the lactational programming hypothesis, they affect metabolic and neurobiological development, and, in consequence, offspring phenotype and behavior [13,14,15,16]. Studies investigated maternal psychological [10,15,17,18,19,20,21,22], socio-demographic [7,10,18,23], or anthropometric [3,10,24] determinants of breastmilk GCs levels provide inconclusive results. Similarly, results regarding the mode of delivery [5,10], preterm birth [6,10,17] or seasonal variations [10,23] were inconsistent.

GCs not only are involved in the stress response but also play a variety of important biological functions, including an improvement of intestinal maturation, microbiome, macronutrients metabolism, growth, body composition, and neurodevelopment [8,25,26]. They may be particularly important during early development, as GCs receptors are found in higher densities in the intestines in infancy and decrease after weaning [13]. Interestingly, it was shown that breastmilk feeding was associated with a 5.5-fold increase in GC expression compared to formula feeding [16]. Moreover, breastfeeding was associated with decreased methylation of the GC receptor gene (NR3C1), and, in turn, was linked to decreased cortisol stress reactivity, which may be responsible for many breastfeeding health benefits [27]. Previous primate and human studies showed that breastmilk cortisol was associated with infant temperament and fear reactivity, but the direction of those associations was inconsistent and sometimes sex-specific [13,15,19,25,26]. Additionally, due to the regulation of macronutrient metabolism and immune system breastmilk, cortisol may affect breastmilk composition [9,21,24,26,28,29], but these results are also unclear, thus further studies are necessary.

The aim of this study was to evaluate breastmilk cortisol levels at 1, 3, and 6 months of lactation, as well as established its associations with: (1) seasonal, maternal socioeconomic, psychosocial, and infant factors; (2) breastmilk macronutrient composition, energy value, and fatty acids profile; (3) infant anthropometric, psychomotor development, and temperament.

## 2. Materials and Methods

### 2.1. Study Design and Data Collection

This observational prospective study conducted in the central area of Poland between 2015–2017 consisted of three study visits during the first, third, and sixth months of lactation. Individuals who were 19 years or older, who gave birth to healthy, at term, single infants no later than 6 weeks, and planned to exclusively breastfeed for 6 months were eligible to participate. More details about the study design and the methods used have been previously described [30,31]. Briefly, data about maternal nutrition were collected using a 3 day food record at the 3rd and 6th months of lactation [31]. Anthropometric measurements in mothers (body weight and height) were conducted according to the ISAK standards [32] using a professional stadiometer (SECA 799, Hamburg, Germany) and analyzed using body mass index according to the WHO [33]. Maternal psychosocial characteristics were assessed at the first, third, and sixth months of lactation using the Polish versions of the Edinburgh Postpartum Scale (EPDS) [34,35] and the Perceived Stress Scale (PSS-10) [36,37]. In the EPDS, 12 points of the total score were the cut-off point for the risk of postpartum depression. Infant anthropometric measurements (weight and length) were measured at the third and sixth months of life using the neonatal scale (SECA 728) and anthropometric tape (SECA 203) according to WHO recommendations [38]. Further, infant BMI and body mass to age, length to age, and BMI to age z-score were calculated using the WHO Anthro Survey Analyzer Software v3.2.2 [39]. A children’s psychologist assessed infant psychomotor development and temperament at the sixth month of life using the Children’s Development Scale DSR [40]. More details about infant psychomotor development were described previously [30].

### 2.2. Participants

**Figure 1:**
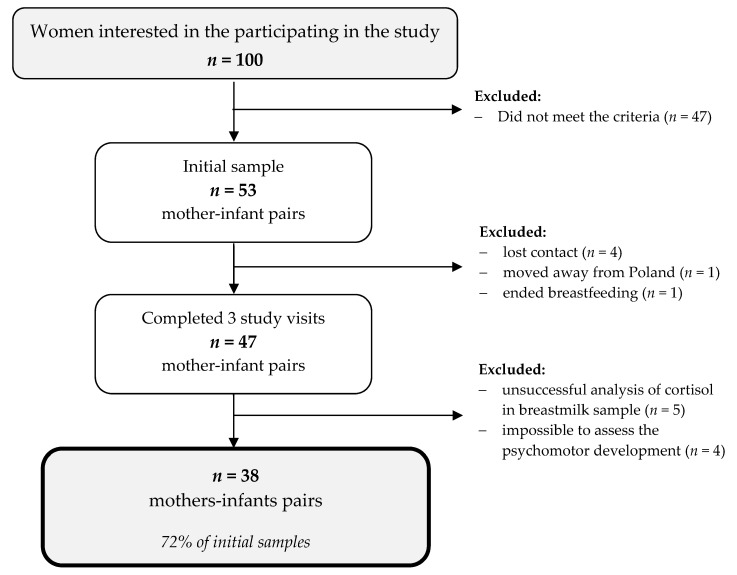
A flowchart of the study sample collection.

Fifty-three mother–infant pairs participated in the study; however, 47 pairs completed all three study visits, and 9 pairs were excluded from the analysis. Thus, in this paper, 38 mother–infant pairs were included (Figure 1).

Mothers included in this analysis were well educated, mostly married, and in a good economic situation, with normal BMI (Table 1). Half of them had given birth to one healthy, male infant with normal development during the first six months of life.

### 2.3. Breastmilk Sample Collection

Mothers who participated in the study collected breastmilk samples at home prior to the study visits at the first, third, and sixth months of lactation according to the received instructions. A sample of 5–10 mL of pre- and post-feeding breastmilk was collected manually or expressed by an electric pump at four time periods (6:00–12:00; 12:00–18:00; 18:00–24:00; 24:00–06:00) into separate polypropylene containers 24 h prior to the study visit. The samples were transported to the laboratory under cooling conditions, and the same amount of each sample was collected and mixed in a Vortex shaker IKA MS2 (IKA Works Inc., Wilmington, NC, USA) for one minute. The pooled sample was stored in 2 mL aliquots in −80 °C for later analysis.

### 2.4. Breastmilk Chemical Analysis

Breastmilk micronutrients and energy value were analyzed using MIRIS human milk analyzer HMA (Miris, Uppsala, Sweden), whereas breastmilk fatty acid profiles were analyzed using gas chromatograph after Folch extraction with modifications as described previously.

For the cortisol analysis, a 2 mL of breastmilk sample was thawed in water at bathroom temperature and sonicated (3 × 10 s, 20 s pause). Then, a 200 µL milk sample was diluted in 200 µL of saline. Breastmilk cortisol levels were determined by using commercial enzyme-linked immunosorbent assay (ELISA) kit (Salivary Cortisol ELISA SLV2930, DRG Instruments GmbH, Marburg, Germany) with the sensitivity of 0.09 ng/mL and detection range of 0.09–30.0 ng/mL. This kit has <0.001% cross-reactivity with testosterone, DHEA-S, estrone (E1), estradiol (E2), 1.47% with estriol (E3), 1.97% with androstenedione, 11.39% with DHEA and 23.40% with progesterone. Analysis was conducted in two parallel repetitions at the Hirszfeld Institute of Immunology and Experimental Therapy. Cortisol values were expressed as ng/mL and the average of the two measurements was used for further statistical analysis.

### 2.5. Statistical Analysis

Statistical analyses were performed with STATISTICA ver. 13.3 (TIBCO Software Inc., Paolo Alto, CA, USA). Data about breastmilk composition, results of EPDS, PSS-10, and DSR scale data were log-transformed prior to statistical analysis. All quantitative data were checked for normality of distribution using the Shapiro–Wilk test and expressed as mean ± standard deviation (SD). Differences in cortisol concentrations between the study visits were assessed using ANOVA for repeated measurement tests, whereas differences between two groups (e.g., season of breastmilk collection) using the Student’s *t*-test. Partial correlations and linear regression analysis were used to assess associations between breastmilk cortisol and maternal and infant factors, as well as breastmilk composition and infant anthropometrics, psychomotor development, and temperament. A *p*-value below 0.05 was considered significant in all the conducted analyses.

## 3. Results

### 3.1. Breastmilk Cortisol Level and Its Determinants

Breastmilk cortisol was stable during the first sixth months of lactation (Table 2). In the first and sixth months of lactation, we observed no consistent significant differences in cortisol according to seasonal variation—in the first month of lactation breastmilk, cortisol levels were higher in the autumn-winter season, whereas they were lower in the sixth month of lactation in the spring-summer season (*p* ≤ 0.05). No significant differences were found according to parity, maternal overweight and obesity, self-assessment of the economic situation and average income, infant sex, and mode of delivery (Table S1).

Breastmilk cortisol levels within the first sixth months of lactation were not correlated to any maternal and infant factors, including maternal psychological characteristics—perceived stress and risk of postpartum depression (Table 3).

### 3.2. Breastmilk Cortisol and Breastmilk Macronutrient and FA Profile

Partial correlation analysis, adjusted for the season of breastmilk sample collection, showed a moderate association between cortisol and crude protein in the third month postpartum, and cortisol and total solids in the sixth month postpartum (Table 3).

Linear regression models, adjusted for season of breastmilk collection, infant age, and number of breastfeedings per day, confirmed associations between breastmilk cortisol and crude protein in the third month of lactation (β = 0.395, 0.084–0.705; Table 4). However, associations with total solids were not confirmed in linear regression analysis.

No significant correlations were observed between breastmilk cortisol and FA profile within the first sixth months of lactation (Table S2).

### 3.3. Breastmilk Cortisol and Infant Anthropometric Development

Partial correlations, adjusted for the season of breastmilk collection, breastmilk energy value, and crude protein, showed that cortisol levels in the third month of lactation were moderately correlated with BMI z-score in the third and sixth months of life in the total group (r = 0.412 and 0.372, *p* ≤ 0.05, respectively). In a separate analysis according to infant sex, those associations were observed only in the third month of life in girls (Table 5).

Associations between breastmilk cortisol in the third month of lactation and infant BMI z-score in the third and sixth months of life were partially confirmed in the multivariate linear regression analysis (Table 6). In the third month of lactation, results were statistically significant after adjusting for average breastmilk energy value (Model 2: β = 0.359, 0.021–0.697).

### 3.4. Breastmilk Cortisol Level and Infant Psychomotor Development and Temperament

Partial correlation analysis controlled for infant sex, age, birthweight, maternal age, education, psychological status, and parity (Table 7) showed moderate adverse associations between breastmilk cortisol in the first and sixth months of lactation and infant regularity in the sixth month of life (r = −0.379 and r = 0.421, respectively, *p* ≤ 0.05). However, separate analyses conducted for boys and girls did not confirm this association. In girls, breastmilk cortisol in the first and sixth months of lactation moderately correlated (r = 0.634 and r = 0.636, respectively, *p* ≤ 0.05) with the centile of performance scale—which reflects psychomotor development. In boys, we observed significant associations between cortisol in the first month of lactation and sensitivity in the sixth month of life (r = 0.580, respectively, *p* ≤ 0.05).

## 4. Discussion

In this prospective study, we showed that breastmilk cortisol levels in the first, third, and sixth months of lactation were unrelated to any maternal or infant factors. We observed that only seasonal variations were associated with cortisol in the breastmilk from the first to six months of lactation, but the observed interactions were inconsistent. Breastmilk cortisol was moderately associated with breastmilk crude protein in the third month of lactation, but associations with other macronutrients and breastmilk FA were not observed. Moreover, in our study, we detected limited associations between breastmilk cortisol in the third month of lactation and infant BMI z-score, but not infant psychomotor development and temperament.

### 4.1. Factors Associated with Breastmilk Cortisol

The cortisol levels observed in our study varied insignificantly from 11.2 to 12.7 ng/mL from the first to the sixth month of lactation. Those results are in line with other authors who reported that cortisol levels were highest in colostrum and relatively stable in transitional and mature milk [4,18]. In contrast, a study from the Netherlands reported that breastmilk cortisol increased from two to 12 weeks of lactation [20,41]. Cortisol levels observed in our study were higher than those observed in other studies [6,10,15,19,21,22,23,24,41,42], but lower than those reported in some studies [7,20,43]. Moreover, we observed significant differences between cortisol levels between the season of sample collection—higher levels of cortisol were observed in samples collected during the autumn-winter season in the first month of lactation, but in the sixth month of lactation in the spring-summer season. Other studies provide inconclusive results—one reported no variations [10], whereas another from Finland showed higher levels in summer [23]. Previously, seasonal cortisol variations in plasma or saliva were reported in regions with clear seasonal variations in light and climate, with the highest levels in spring [44,45], summer [46], or fall/winter [47]. Discrepancies between studies assessing breastmilk cortisol may be related to variations in cortisol analysis methods, as in immunoassays, other steroids may cross-react and LC-MS/MS is the most specific [8,42]. However, the results observed in our study were lower than the results obtained by both methods [7,20,43]. Another possible explanation for those differences in sample collection was that glucocorticosteroids in breastmilk reflect HPA axis activity with a peak early in the morning [6,7,8,20,22]. Breastmilk samples in our study were collected four times per day to minimize diurnal variations in breastmilk cortisol, but morning samples could be collected between 6:00 a.m. and 12:00 p.m.

#### 4.1.1. Maternal Factors

In our group, breastmilk cortisol across the first six months of lactation was unrelated to maternal age, self-assessed economic situation, and average income per capita—which is in line with previous studies [10,18,23]. Interestingly, previous studies showed that low maternal education was significantly associated with higher cortisol levels [10,23], which confirms that education plays an important role in individual stress response [48]. However, the mothers who participated in our study were very well-educated, so we were unable to explore these associations. According to previous human [23] and primate [26] studies, primiparas compared to multiparas mothers have higher cortisol levels—probably due to differences in the mammary gland physiology [26]. However, our study and studies by Pundir et al. [7,10] did not confirm these results. Moreover, maternal pre-pregnancy BMI was associated with higher cortisol levels [24], and other studies reported higher cortisone—but not cortisol levels—in mothers with normal weight mothers compared to underweight or overweight [10]. However, our results and those by Hinde et al. [26] from rhesus monkeys also did not confirm associations between maternal BMI and breastmilk cortisol.

Many previous studies tried to explore the associations between maternal psychological characteristics—especially depression or stress—on cortisol levels in breastmilk [10,15,18,19,20,21,22,23], saliva [12,28], serum [12,43], hair [49], and urine [12]. It was reported that maternal hostility [18] and breastfeeding satisfaction [17] were positively correlated with breastmilk cortisol. However, the majority of those studies reported no associations between stress or anxiety and breastmilk [10,15,19,21], saliva [22,28], and hair [49] cortisol, which was in line with our observations based on breastmilk cortisol. On the contrary, Aparicio et al. [20] reported higher breastmilk in mothers with increased stress and anxiety but not depressive symptoms. Similarly to Aparicio et al. [20], we and other researchers did not observe a significant association between maternal depression and breastmilk [10,15,19,21,23,43], serum [12,43], saliva [12], hair [49], and urine [12] cortisol. However, Romjin et al. [22] reported that patients in a psychiatry-obstetric-pediatric clinic had a lower milk cortisol area under the curve compared to controls and that breastmilk and saliva cortisol was highly correlated, but no difference in glucocorticosteroid rhythmicity was observed. Those results indicated that maternal stress did not affect cortisol transport into the mammary gland [22]. The predominant lack of any associations between maternal stress or depression and breastmilk cortisol may be related to several factors. Firstly, large variability in the study design (including the characteristics of the study group), the methods used, and outcomes of maternal psychological state may lead to differences in the sensitivity of the method and, in consequence, hamper detecting associations and the comparison of results [12,20,22,23]. Secondly, during acute stress, the HPA axis is triggered, which leads to elevation of glucocorticosteroid levels; however, chronic stress may lead to the exhaustion of the HPA axis related to a decrease in glucocorticosteroid levels and responsiveness to stressors [12,22].

#### 4.1.2. Infant- and Delivery-Related Factors

In our study, we did not observe any associations or differences in breastmilk cortisol in terms of infant sex, birthweight, gestational age, or mode of delivery—which is in line with previous studies. Studies among humans [7,19,23] and rhesus macaques [26] reported no differences according to infant sex, with one exception, where mothers of male rhesus had higher cortisol levels in the milk but not plasma [13]. Results regarding mode of delivery provided contradictory results—in one study, differences were not observed [10], whereas another study reported differences in cortisol changes in the postpartum period and higher correlations to plasma after cesarean delivery [5].

### 4.2. Breastmilk Cortisol and Breastmilk Composition

Glucocorticosteroids are crucial for lactogenesis and contribute to nutrient metabolism and the regulation of a variety of physiological processes in the mammary gland, including protein synthesis, nutrient flux, prevention of cell death in the mammary gland, and milk yield [7,26,50,51,52,53]. Thus, breastmilk cortisol levels may affect breastmilk composition of macronutrients, the fatty acid profile, or immune factors. Previous studies reported associations with secretory immunoglobin A (sIgA) and breastmilk cortisol [17,18], but recent studies did not confirm associations between salivary [29] or breastmilk cortisol [20] and sIgA, as well as other immunological components of breastmilk. In our study, we observed a positive association between breastmilk cortisol and crude protein at three months of lactation—which was significant after adjustment for the season of breastmilk collection, infant age, and the number of breastfeedings. These results are in line with studies on rhesus monkeys [13,26], but other human studies did not confirm these observations [9,29]. Moreover, primate studies showed positive associations also to breastmilk fat [13,26], and human studies showed positive associations between fat and salivary cortisol [28] and negative associations between lactose and salivary [28] or cord blood [54] cortisol. We also explored associations between breastmilk cortisol and breastmilk FA profile, but we did not observe any significant correlations at any lactation stage. Previous studies reported higher levels of lauric and myristic FA in the breastmilk samples with high breastmilk cortisol levels [24]. In another study, breastmilk long chain fatty acids, monounsaturated fatty acids, and polyunsaturated fatty acids were positively associated with salivary cortisol but not maternal stress, whereas medium chain fatty acids were related only to maternal stress, not cortisol levels [28].

Previous human and dairy animal studies showed that cortisol may affect protein, glucose, and lipid metabolism and, in consequence, increase the availability of glucose and fatty acids, which are an essential precursor for milk production [50,55,56,57,58]. An in vivo study confirmed that cortisol regulates protein metabolism in the mammary epithelial cells [52], which explains the positive association observed in our study. Moreover, this association was time-dependent, as we observed it at the third, but not first and sixth months of lactation. Previous animal studies showed that cortisol’s effect on milk yield may be dependent on the lactation stage [53], but it was not shown in studies regarding milk composition, and the majority of studies were short-term or cross-sectional [9,13,26,28,51]. Our study did not confirm the influence of cortisol on milk lipids, despite that the influence of cortisol on lipolysis, the release of medium and intermediate chain FA, and the synthesis of long-chain FA had been previously shown [24,58,59,60]. A possible explanation for the discrepancies between the studies could be differences in the method of sample collection. We collected fore- and hindmilk samples multiple times per day, whereas in other human studies only foremilk from all feedings during the day [9] or milk from the full breast between the second and third feedings [28] was collected. Moreover, it was shown that the synthesis of different nutrients in the mammary gland is regulated independently and multidirectional [51,54,61], which may explain why we did not detect any associations with lactose and fat. Furthermore, this may indicate that macronutrients and fatty acids are transported into milk independently of cortisol [9].

### 4.3. Breastmilk Cortisol and Infant Anthropometric Development

We also investigated associations between breastmilk cortisol and infant anthropometric development. We found a positive association between breastmilk cortisol in the third month of lactation and infant BMI z-score in the third and sixth months of lactation; but after adjustment for infant birthweight and sex, those associations were no longer significant. Our results are in line with the study from the Netherlands, where the authors found no associations between diurnal cortisol rhythm in the first month and infant growth and body composition at the third month of lactation [9]. Furthermore, a study from the USA found a negative effect between breastmilk cortisol at the third month of lactation and BMI percentile gain in the first two years of life, and this influence was stronger in girls than boys [62]. However, in this study, only a single breastmilk sample was collected between 11:30 a.m. and 4:00 p.m., which may result in high variability and may be insufficient to analyze cortisol transmission via breastmilk [63]. Interestingly, the primate study by Hinde et al. [26] reported associations between greater daily growth and higher cortisol during lactation peak or lower cortisol and higher available milk energy at early lactation. Possible discrepancies between studies may be a result of differences in sample collection (both number of samples and timing of collection) as well as cortisol analysis methods. A recent meta-analysis of associations found a significant association between hair (but not serum or salivary) cortisol and children and BMI z-score; however, only in studies extracting cortisol via more sensitive and reliable LC-MS/MS, and not ELISA [64]. However, Hollanders et al. [9] used the LC-MS/MS method, but also did not observe significant associations. Probably, the small sample size in the Hollanders et al. [9] and our study (*n* = 42 and *n* = 38, respectively), as well as the short duration of observation (3 and 6 months, respectively), was too small to detect significant associations that may be noticeable in further infant development. Animal studies suggest that cortisol improves intestinal development—including the microbiome and intestinal immune system—which in consequence may affect anthropometric development [8,63,65].

### 4.4. Breastmilk Cortisol and Infant Psychomotor Development and Temperament

Cortisol may also affect the brain and, in consequence, cognitive and behavioral development [13,15,16,18,19,25,26,32,66]. Thus, in our study, we also explored the associations between breastmilk cortisol and infant psychomotor development and temperament in the sixth month of life. In the total group, we observed negative associations between regularity in the biological and behavioral patterns and breastmilk cortisol in the first and sixth months of lactation. Moreover, in girls, we detected a positive association between psychomotor development and breastmilk cortisol in the first and sixth months of lactation; whereas, in boys, we detected positive associations between sensory sensitivity and breastmilk cortisol in the first month of lactation. However, those gender- and time-specific associations were only significant in partial correlations analysis adjusted for infant sex (in the total group) and age, birthweight maternal age, education, psychological status, and parity. Previous human and animal studies showed that breastmilk or plasma cortisol may be associated with negative behavioral outcomes, such as nervous and less confident temperament [26], higher fear or negative reactivity [15,19,25], impulsivity on the cognitive task [16], and some of the studies found this only in girls [15,19]. Some positive associations were also observed between cortisol and infant neurobiological development and temperament. Studies on rhesus macaques found that higher breastmilk cortisol was related to a more confident temperament in sons [13] and frequency of play in girls, and human studies found a positive association with newborn homeostatic adjustments of the nervous system and better capacity to control involuntary responses (changes in skin color, tremors, and startles) [18]. However, in two human studies, no associations with behavior or sleep pattern [66] or infant crying [41] were found. Rodent studies confirmed associations between maternal cortisol and cognitive and behavioral development. However, cortisol also had a positive impact on fear behavior and cognitive and motor development, contrary to human studies [67,68,69,70]. Discrepancies between studies may be related to interspecies differences as well as variability in study design, including the timing of outcomes assessment, such as gender- and time-specific glucocorticosteroid sensitivity [16,25,26,41]. Moreover, different methods of cognitive, behavior, and temperament assessment were used and these may have a different sensitivity, which could hamper the detection of associations [31].

### 4.5. Strengths and Limitations

Our study has several strengths. First, the study analyzed breastmilk samples obtained from three time points over the first six months of lactation: first, third, and sixth month. Second, the analyzed breastmilk samples contained both fore- and hindmilk in equal proportions. Third, mothers collected four samples per study point (6:00–12:00; 12:00–18:00; 18:00–24:00; 24:00–6:00), which decreased the influence of diurnal variations in the HPA axis [6,7,8]. However, we pooled the same amount of each sample, so the specific analysis of diurnal variations in cortisol and the calculation of an area under the curve was impossible. Fourth, we analyzed breastmilk macronutrients and FA profiles. Fifth, we collected quite extensive data on mother–infant pairs who participated in the study. Sixth, we assessed infant anthropometric parameters at two time points using the WHO standards.

However, our study is not without limitations. First, our study sample was relatively small. Second, our sample was very well educated and with higher socioeconomic status than the national average, as well as breastfeeding for a longer period [71]. This may be related to self-selection bias and results with non-participating mothers with poorer psychological status; thus, some effects could not be detected, and results should be generalized with caution. Third, we assessed only breastmilk cortisol, which is more active than cortisone and has direct links with metabolism and neurodevelopment but occurs in breastmilk in lower concentrations. Fourth, we analyzed cortisol using the ELISA method, which is less specific compared to the LC-tandem MS assay, but our results were comparable to some studies using this method [7,42].

## 5. Conclusions

In conclusion, this study found limited associations between breastmilk cortisol and seasonal factors, and breastmilk crude protein and infant BMI z-score. On the other hand, we did not confirm associations between breastmilk cortisol and maternal socioeconomic, psychosocial, infant factors, or other breastmilk macronutrients, energy value, fatty acid profile, and infant psychomotor development and temperament. Our results support most previous human studies. Hence, further follow-up studies on a large mother–infant pair population with serial cortisol measurements over a longer period of time are necessary.

## Figures and Tables

**Table 1:** Characteristics of mother–infant pairs participating in the study.

Variable	Mean ± SD	Min–Max	*n* (%)
Maternal sociodemographic factors			
Maternal age (years)	30.9 ± 3.7	22.7–40.0	
Parity, primiparous			19 (50)
University education			38 (100)
Marital status, married			32 (84)
Self-assessment of the economic situation, very good			14 (37)
Average monthly income, >1500 PLN per capita			25 (66)
Maternal biological and psychological factors			
Gestational weight gain (kg)	13.5 ± 4.4	0.7–22.0	
BMI, pre-pregnancy (kg/m^2^); overweight or obesity	22.7 ± 3.7	18.4–38.6	5 (13)
BMI, 1st visit (kg/m^2^); overweight or obesity	24.0 ± 3.9	18.4–39.7	9 (24)
BMI, 2nd visit (kg/m^2^); overweight or obesity	23.5 ± 4.1	18.1–40.8	6 (16)
BMI, 3rd visit (kg/m^2^); overweight or obesity	23.1 ± 4.3	19.1–42.6	7 (18)
EPDS, 1st visit (points); depression risk	5.9 ± 4.0	0–18	3 (8)
EPDS, 3rd visit (points); depression risk	4.7 ± 3.7	0–18	2 (5)
EPDS, 6th visit postpartum (points); depression risk	5.2 ± 4.1	0–19	3 (8)
PSS-10, 1st visit (points); high stress	21.0 ± 5.1	4–28	23 (61)
PSS-10, 2nd visit (points); high stress	20.4 ± 3.8	13–28	22 (58)
PSS-10, 3rd visit (points); high stress	21.5 ± 4.0	14–29	23 (61)
Infant and breastfeeding factors			
Infant gender, boys			19 (50)
Infant age, 1st visit (weeks)	4.8 ± 0.8	3.4–6.9	
Infant age, 2nd visit (weeks)	13.4 ± 1.7	9.6–20.1	
Infant age, 3rd visit (weeks)	26.9 ± 1.2	24–29.6	
Gestational age (Hbd)	39.3 ± 1.3	37–42	
Mode of delivery, C section			16 (42)
Birthweight (g)	3413.9 ± 373.7	2870–4240	
BMI z-score, 2nd visit	−1.15 ± 1.48	−4.17–3.82	
BMI z-score, 3rd visit	−1.31 ± 1.32	−4.17–1.24	
Children Development Scale (DSR) Results			
Performance Scale, Manipulation (points); average	6.4 ± 1.1	3–7	28 (74)
Performance Scale, Perception (points)	4.9 ± 1.1	2–7	
Performance Scale, Memory (points)	0.8 ± 0.4	0–1	
Performance Scale, Speech and language (points)	2.1 ± 1.0	1–5	
Performance Scale, Social behavior (points); high	6.7 ± 1.0	4–9	3 (8)
Performance Scale, Motor skill (points); high	4.1 ± 1.1	2–6	15 (39)
Performance Scale, total result (centiles); elevated	40.4 ± 13.8	16–74	2 (5)
Observational Scale, Vigor (points); high	10.6 ± 1.2	9–13	3 (8)
Observational Scale, Adaptability (points); high	13.7 ± 1.8	7–17	14 (36)
Observational Scale, Regularity (points); high	3.3 ± 0.8	1–7	2 (5)
Observational Scale, Sensitivity (points); high	3.4 ± 0.5	3–4	13 (34)

BMI—body mass index; EPDS—Edinburgh Postpartum Depression Scale; Hbd—hebdomas; weeks of gestation; PSS-10—Perceived Stress Scale.

**Table 2:** Breastmilk cortisol at 1, 3, and 6 months postpartum according to the season of breastmilk collection.

	Breastmilk Cortisol [ng/mL]		
	Mean ± SD		
	Min–Max		
	1 Month Postpartum	3 Months Postpartum	6 Months Postpartum
**Total group (*n* = 38)**	11.2 ± 6.2	11.2 ± 4.3	12.7 ± 6.2
	5.0–32.0	4.8–23.6	4.4–29.4
***p*-value ^1^**	0.154		
**Season of breastmilk sample collection**			
spring-summer	*n* = 19	*n* = 24	*n* = 21
	9.0 ± 3.6	11.9 ± 5.0	14.3 ± 5.9
	5.0–16.4	4.8–23.6	5.0–29.4
	*n* = 19	*n* = 14	*n* = 17
autumn-winter	13.5 ± 7.5	9.9 ± 2.6	10.8 ± 6.1
	5.8–32.0	5.0–14.2	4.4–26.0
***p*-value ^2^**	0.020	0.332	0.041

^1^ ANOVA for repeated measurements test; ^2^ Student’s *t*-test. Tests were conducted on log-transformed cortisol data.

**Table 3:** Partial correlations between breastmilk cortisol (log), maternal and infant factors, and breastmilk macronutrient composition, adjusted for season of breastmilk sample collection.

	Variable	Breastmilk Cortisol (Log)		
		1 Month Postpartum	3 Months Postpartum	6 Months Postpartum
Maternal factors	Maternal age	0.028	0.008	−0.305
	Maternal BMI	−0.091	0.040	−0.081
	EPDS score	0.262	0.090	−0.055
	PSS-10 score	0.299	0.026	−0.038
Infant factors	Infant age	−0.182	−0.001	0.030
	Gestational age	0.186	0.063	0.258
	Birthweight	−0.107	0.111	0.077
	Number of breastfeedings	0.147	−0.166	0.086
Breastmilk composition	Fat	0.005	−0.016	0.212
	Protein	0.081	0.281	0.151
	Carbohydrates	0.081	0.013	0.258
	Total solids	−0.092	−0.001	0.358 *
	Crude protein	−0.080	0.366 *	0.126
	Energy	−0.089	0.011	0.282

BMI—body mass index; EPDS—Edinburgh Postpartum Depression Score; PSS-10—Perceived Stress Scale. * *p* ≤ 0.05.

**Table 4:** Linear regression models between breastmilk cortisol(log) and breastmilk crude protein in the first sixth months lactation.

Breastmilk Crude Protein	Breastmilk Cortisol(log)		
	Model 1	Model 2	Model 3
1 month lactation	β = −0.013 (−0.351–0.325) R^2^= −0.03; *p* = 0.940	β = 0.022 (−0.331–0.376) R^2^= 0.07; *p* = 0.143	β = −0.011 (−0.434–0.412) R^2^ = −0.02; *p* = 0.518
3 months lactation	β = 0.395 (0.084–0.705) ** R^2^ = 0.13; *p* = 0.014	β = 0.311 (0.021–0.601) * R^2^= 0.27; *p* = 0.002	β = 0.318 (0.007–0.630) * R^2^ = 0.19; *p* = 0.030
6 months lactation	β = 0.150 (−0.184–0.484) R^2^ = −0.01; *p* = 0.369	β = 0.133 (−0.232–0.498) R^2^ = −0.06; *p* = 0.830	β = 0.108 (−0.273–0.489) R^2^ = −0.08; *p* = 0.882

Model 1: unadjusted; Model 2: adjusted for the season of breastmilk collection and infant age; Model 3: model 2 adjusted for the number of breastfeedings. * *p* ≤ 0.05; ** *p* ≤ 0.01.

**Table 5:** Partial correlations between breastmilk cortisol(log) and infant BMI z-score at the 3rd and 6th months adjusted for the season of breastmilk collection and breastmilk energy value and crude protein.

	Variable	Breastmilk Cortisol (Log)		
		1 Month Postpartum	3 Months Postpartum	6 Months Postpartum
Total	3 months	−0.052	0.412 *	−0.077
	6 months	0.094	0.372 *	−0.004
	∆ 3–6 months	−0.187	−0.079	0.097
Girls	3 months	0.005	0.541 *	-
	6 months	−0.121	0.388	0.021
	∆ 3–6 months	−0.165	−0.204	−0.385
Boys	3 months	−0.002	0.371	-
	6 months	0.141	0.288	0.088
	∆ 3–6 months	0.184	−0.183	0.395

∆ 3–6 months—changes in the BMI z-score between the 6th and 3rd months of life. * *p* ≤ 0.05.

**Table 6:** Linear regression models between breastmilk cortisol(log) and infant anthropometric development.

	BMI z-Score Month	Model 1	Model 2	Model 3	Model 4
**cortisol_log_** 1 month	3	β = 0.061	β = 0.081	β = −0.081	β = −0.071
		(−0.403–0.282)	(−0.429–0.268)	(−0.388–0.277)	(−0.395–0.253)
		R^2^ = −0.02	R^2^ = −0.02	R^2^ = 0.21 *	R^2^ = 0.18 *
	6	β = 0.094	β = 0.104	β = 0.096	β = 0.018
		(−0.253–0.441)	(−0.260–0.468)	(−0.262–0.454)	(−0.346–0.382)
		R^2^ = −0.02	R^2^ = −0.05	R^2^ = 0.02	R^2^ = 0.02
**cortisol_log_** 3 month	3	β = 0.338	β = 0.359	β = 0.265	β = 0.286
		(0.015–0.661) *	(0.021–0.697) *	(−0.046–0.577)	(−0.034–0.607)
		R^2^ = 0.09 *	R^2^ = 0.10 *	R ^2^ = 0.27 **	R^2^ = 0.26 **
	6	β = 0.337	β = 0.451	β = 0.405	β = 0.334
		(0.009–0.665) *	(0.095–0.808) **	(0.029–0.780) *	(−0.065–0.733)
		R^2^ = 0.09 *	R^2^ = 0.11	R^2^ = 0.11	R^2^ = 0.11
**cortisol_log_** 6 months	6	β = 0.032	β = 0.002	β = 0.00	β = −0.006
		(−0.380–0.317)	(−0.363–0.367)	(−0.359–0.359)	(−0.355–0.343)
		R^2^ = −0.03	R^2^ = −0.07	R^2^ = −0.03	R^2^ = 0.03

BMI—body mass index. Model 1: unadjusted; Model 2: adjusted for average breastmilk energy value; Model 3: model 2 adjusted for birthweight; Model 4: model 3 adjusted for infant sex. * *p* ≤ 0.05; ** *p* ≤ 0.01.

**Table 7:** Partial correlations between breastmilk cortisol (log) and infant psychomotor development and temperament at the 6th month of life adjusted for infant sex (in the total group) and age, birthweight maternal age, education, psychological status, and parity.

	DSR Scale	Breastmilk Cortisol (Log)		
		1 Month	3 Months	6 Months
Total	Manipulation	0.296	0.269	0.184
	Perception	0.169	−0.132	−0.160
	Memory	−0.213	−0.144	−0.013
	Speech	0.057	0.089	0.005
	Social behavior	−0.049	0.162	−0.085
	Motor	−0.219	0.053	−0.057
	Performance scale score	0.069	0.144	−0.035
	Performance scale centile	0.230	0.282	0.117
	Vigor	−0.011	0.101	−0.011
	Adaptability	−0.235	0.138	−0.214
	Regularity	−0.379 *	0.038	−0.421 *
	Sensitivity	0.169	−0.016	0.071
Girls	Manipulation	0.341	0.090	0.111
	Perception	0.385	−0.448	−0.109
	Memory	−0.025	−0.079	0.425
	Speech	0.468	0.229	0.425
	Social behavior	−0.370	−0.054	−0.139
	Motor	0.017	0.538	0.284
	Performance scale score	0.403	0.144	0.369
	Performance scale centile	0.634 *	0.428	0.636 *
	Vigor	0.060	0.419	−0.148
	Adaptability	−0.017	0.299	−0.231
	Regularity	−0.353	0.088	−0.276
	Sensitivity	−0.121	−0.344	0.047
Boys	Manipulation	−0.084	0.100	0.139
	Perception	−0.309	−0.197	−0.351
	Memory	−0.313	−0.030	−0.555
	Speech	0.253	0.219	−0.227
	Social behavior	−0.070	0.434	−0.348
	Motor	−0.275	0.119	−0.363
	Performance scale score	−0.291	0.256	−0.325
	Performance scale centile	−0.237	0.369	−0.399
	Vigor	0.244	0.167	−0.046
	Adaptability	−0.127	0.486	−0.241
	Regularity	−0.428	0.207	−0.173
	Sensitivity	0.580 *	−0.016	−0.058

DSR—Children’s Developmental Scale. * *p* ≤ 0.05.

## Data Availability

The data presented in this study are available on request from the corresponding author.

## Supplementary Materials

The following supporting information can be downloaded at: https://www.mdpi.com/article/10.3390/ijerph192214809/s1, Table S1: Breastmilk cortisol at 1, 3, and 6 months postpartum according to maternal parity, infant sex, mode of delivery, and season of breastmilk collection. Table S2: Partial correlations between the breastmilk cortisol (log) and breastmilk fatty acid profile (log) adjusted for season of breastmilk collection.
