# Supplementary material for: Factors Influencing Cortisol Concentrations in Breastmilk and Its Associations with Breastmilk Composition and Infant Development in the First Six Months of Lactation
Source: Int J Environ Res Public Health. 2022 Nov 10;19(22):14809. doi: 10.3390/ijerph192214809 (PMC9690377; doi:10.3390/ijerph192214809)
Supplement: Supplementary file 1 [file ijerph-19-14809-s001.zip › ijerph-2024012-supplementary.pdf]

International Journal of
Environmental Research and Public Health

MDPI

Article

Factors Influencing Cortisol Concentrations in Breastmilk and Its Associations with Breastmilk Composition and Infant Development in the First Six Months of Lactation

Monika A. Zielinska-Pukos, Joanna Bryś, Natalia Kucharz, Agnieszka Chrobak, Aleksandra Wesolowska, Iwona Grabowicz-Chądrzyńska and Jadwiga Hamulka

Table S1. Breastmilk cortisol at 1, 3, and 6 months postpartum according to maternal parity, infant sex, mode of delivery, and season of breastmilk collection.

	Breastmilk cortisol [ng/ml]<br>Mean ± SD<br>min – max		
	**1 month postpartum**	**3 months postpartum**	**6 months postpartum**
**Parity**			
primiparous (*n*=19)	10.6 ± 6.9<br>5.0 – 32.0	10.5 ± 3.6<br>5.2 – 16.2	11.7 ± 4.9<br>4.4 – 22.2
multiparous (*n*=19)	11.9 ± 5.6<br>5.0 – 25.0	11.8 ± 5.0<br>4.8 – 23.6	13.7 ± 7.2<br>4.8 – 29.4
**p-value** [1]	0.549	0.350	0.313
**Maternal BMI**			
normal	*n*=29<br>10.9 ± 5.8<br>5.0 – 32.0	*n*=32<br>11.1 ± 3.8<br>4.8 – 22.0	*n*=31<br>13.0 ± 6.2<br>4.4 – 29.4
overweight and obesity	*n*=9<br>12.3 ± 7.9<br>5.0 – 25.0	*n*=6<br>11.6 ± 6.9<br>5.0 – 23.6	*n*=7<br>11.3 ± 6.0<br>5.6 – 20.8
**p-value** [1]	0.560	0.793	0.528
**Self-assessment of economic situation**			
good or lower (*n*=24)	10.3 ± 5.6<br>5.0 – 25.0	10.8 ± 4.2<br>5.0 – 23.6	12.7 ± 6.4<br>4.8 – 29.4
very good (*n*=14)	12.8 ± 7.1<br>6.8 – 32.0	11.8 ± 4.6<br>4.8 – 22.0	12.8 ± 5.9<br>4.4 – 26.0
**p-value**[1]	0.247	0.488	0.954
**Average income**			
≤1500 PLN (*n*=13)	8.7 ± 3.2<br>5.0 – 16.4	10.6 ± 3.8<br>5.0 – 16.2	11.6 ± 7.3<br>4.4 – 29.4
>1500 PLN (*n*=25)	12.6 ± 7.0<br>5.0 – 32.0	11.5 ± 4.6<br>4.8 – 23.6	13.3 ± 5.6<br>5.6 – 26.0

**p-value[1]**	0.069	0.551	0.418
**Infant sex**			
boy(n=19)	12.7 ± 7.2<br>5.00 – 32.0	12.2 ± 5.1<br>5.2 – 23.6	12.2 ± 5.1<br>5.0 – 22.2
girl (n=19)	9.8 ± 4.9<br>5.0 – 25.0	10.1 ± 3.2<br>4.8 – 15.2	13.2 ± 7.2<br>4.4 – 29.4
**p-value[1]**	0.151	0.148	0.634
**Mode of delivery**			
C-section (n=16)	11.8 ± 7.0<br>5.0 – 25.0	10.7 ± 4.4<br>5.0 – 23.6	12.6 ± 5.3<br>4.8 – 22.2
vaginal (n=22)	10.8 ± 5.7<br>5.2 – 32.0	11.5 ± 4.4<br>4.8 – 22.0	12.8 ± 6.8<br>4.4 – 29.4
**p-value[1]**	0.641	0.582	0.945

[1] Student’s T-test. Tests were conducted on log-transformed cortisol data.

Table S2. Partial correlations between the breastmilk cortisol(log) and breastmilk fatty acid profile(log) adjusted for season of breastmilk collection.

Breastmilk fatty acid (% of FA(log))	Breastmilk cortisol(log)		
	1 month	3 months	6 months
% SFA	-0.083	-0.046	-0.072
C8:0	-0.239	-0.100	-0.232
C10:0	-0.258	-0.032	0.041
C12:0	-0.171	0.158	0.127
C14:0	-0.008	0.071	0.193
C15:0	0.005	-0.222	-0.081
C16:0	-0.005	-0.117	-0.233
C17:0	-0.041	-0.081	-0.197
C18:0	0.020	-0.050	-0.069
C20:0	0.190	-0.131	0.162
% MUFA	0.170	0.102	0.104
C14:1	-0.033	-0.212	-0.111
C15:1	0.052	-0.154	-0.042
C16:1	-0.050	0.167	-0.323
C17:1	0.015	0.029	-0.262
C18:1	0.173	0.090	0.138
C20:1	0.164	0.248	0.186
% PUFA	-0.031	-0.082	0.023
% n-6 PUFA	-0.035	-0.065	0.013
C18:2 n-6 LA	-0.044	-0.083	0.013
C20:2	0.081	0.277	0.153
C18:3	0.120	-0.008	0.051
C20:3	0.101	0.084	-0.069
C20:4 n-6 AA	0.104	-0.053	-0.056
% n-3 PUFA	-0.020	-0.105	0.027
C18:3 n-3 ALA	0.050	-0.056	-0.032
C20:5 n-3 EPA	0.102	0.262	0.177
C22:6 n-3 DHA	-0.042	-0.178	0.102
Total trans	0.009	-0.296	-0.023
C18:1 trans	0.025	-0.288	-0.127
C18:2 trans	-0.003	-0.180	-0.009

AA – arachidonic acid; ALA - α-linolenic acid; DHA – docosahexaenoic acid; EPA – eicosapentaenoic acid; LA – linoleic acid; MUFA – monounsaturated fatty acids; PUFA – polyunsaturated fatty acids; SFA – saturated fatty acids.
